# Navigating the politics of transformational adaptation in international climate negotiations

**DOI:** 10.1038/s44168-025-00279-6

**Published:** 2025-07-25

**Authors:** Robbert Biesbroek, Emilie Broek, Dore Engbersen, Max van Deursen, Rohan Agarwal, Adelle Thomas, Aditi Mukherji, Lucy Njuguna

**Affiliations:** 1 Public Administration and Policy group, Wageningen University and Research, Wageningen, The Netherlands; 2 Environmental Policy group, Wageningen University and Research, Wageningen, The Netherlands; 3 Natural Resources Defense Council (NRDC), New York, NY USA; 4 Climate Change Adaptation and Mitigation Impact Action Platform, Consultative Group on International Agricultural Research (CGIAR), Nairobi, Kenya; 5 Climate Action, International Center for Tropical Agriculture (CIAT), Nairobi, Kenya

**Keywords:** Governance, Climate-change adaptation, Climate-change policy, Politics

## Abstract

Transformational adaptation to climate risks is an emerging topic in international climate negotiations. However, political views diverge on the desirability and feasibility of introducing transformational adaptation as a new concept. While scientific efforts to clarify its meaning are necessary, only by critically reflecting on the political nature of the concept can the negotiations move forward.

The calls for transformational adaptation have been loud and widespread in recent years; scientists, policy makers, and civil society recognize that the current depth, scope and pace of adaptation is not enough to keep up with the rate of accelerating climate change risks^1,2^. Some are calling for transformational adaptation in order to go beyond ‘incremental’ or ‘business-as-usual’ adaptation to close the growing adaptation gap. Where incremental adaptation refers to minor changes to existing systems, at its core, transformational adaptation is about fundamentally changing systems to address the root causes that drive societal and ecological vulnerability. Despite its popularity, the concept of transformational adaptation faces considerable political contestation, particularly at the international level. It is important to unpack the political nature of transformational adaptation in these increasingly politicized arenas by exploring the main arguments for and against it and identifying pathways to navigate future negotiations.

## From a scientific niche concept to a prominent role in international climate politics

Transformational change has a longstanding history in various fields of social sciences, but it took until the early 2010s before several seminal papers^3,4^ took up the concept in the context of responding to climate risks. Although the need for transformational change was already recognized in the IPCC fifth assessment cycle, it became more central in the sixth assessment cycle, when the IPCC provided a definition of transformational adaptation, and it featured frequently in the Working Group II report^1^. Recently, academic literature on transformational adaptation has rapidly expanded, offering a range of conceptual frameworks, indicators, barriers, enablers, and examples of cases from around the world^5–9^. Yet, what constitutes ‘transformational adaptation’ is a topic of continued academic debate.

The growing academic calls for transformational adaptation, particularly by the IPCC, have propelled the concept from mostly a scientific discourse into the international political arena. The COP28 decision on the Global Goal on Adaptation in 2023, for example, called upon the need to strengthen long-term transformational and incremental adaptation^10^ and requested the UNFCCC secretariat to scope understandings and assessment of transformational adaptation. In November 2024, the technical report prepared by the secretariat on defining and understanding transformational adaptation^11^, offered a rich description but was considered too late and technical to inform political debates at COP29. During COP29, the importance of both incremental and transformational adaptation was again recalled but not elaborated, largely due to different views on what it entails and the consequences of its implementation.

The rise of transformational adaptation comes at a time of growing political attention to adaptation more generally, including through the Baku Adaptation Road Map adopted at COP29. The roadmap, which is still being developed, aims to elevate the political side and implementation of adaptation, and has been proposed by some countries as a catalyst for high-level dialogue and knowledge exchange over the concept. This political attention towards adaptation is also occurring in other negotiation streams of the UNFCCC, including in relation to the new quantified goal on climate finance (NCQG), the update of the NAP technical guidelines, the Loss and Damage mechanism, and the second Global Stocktake.

Currently, however, the political debates on transformational adaptation are hardly moving forward as there are contrasting perspectives on its definition, importance, and implications.

## Different perspectives on transformational adaptation

Drawing on our engagement with these political debates and exploring publicly available documentation of the COP28-29 negotiations through the UNFCCC portal and the Earth Negotiations Bulletin, we identify several frequently heard arguments on transformational adaptation used in the international arenas.

### Arguments for embracing the concept

Those who typically embrace the concept stress the importance of ensuring long-term climate resilience. Globally, progress on adaptation is slow, and more accelerated and fundamental changes through transformational adaptation are urgently needed to avoid disastrous impacts in the future. This is particularly true in a warmer world where current adaptation is much less effective. Transformational adaptation is thus about raising the ambition to do more, better, quicker, and with longer-term futures in mind.

A second argument builds on this. It highlights the critical role of science-based guidance, particularly from the IPCC, in supporting domestic policies for climate action. The scientific community increasingly emphasizes that incremental measures are insufficient to close the growing adaptation gap and calls for transformational adaptation to address the scale and urgency of climate risks. Integrating the latest science into national policy design, planning, implementation and evaluation—including transformational adaptation - is therefore essential for effective and forward-looking adaptation action.

A final argument, but less explicitly voiced, is to make sure that multilateral climate finance is invested ‘wisely’; in other words, avoiding investment decisions that are inefficient, or may increase risks over time, or displace risks to other regions or vulnerable groups. In this view, transformational adaptation does not restrict investing in incremental adaptation where needed, nor should it be seen as a condition for supporting implementation. For some countries, calls for transformational adaptation might actually be ways to increase their international finance and support to better address their severe and existential threats from climate change.

### Arguments for questioning the concept

At the same time, various concerns have been voiced about considering transformational adaptation in the international negotiations.

First, almost all countries refer to the lack of a universal definition and ambiguity around the concept. The IPCC definition is considered too broad and generic, offering few actionable perspectives. There are also limited concrete examples of transformational adaptation documented, and thus, it is unclear what it entails in practice. For example, in many rural areas of low and middle income countries without basic health or sanitation services, introducing these services can be transformational. However, where such facilities already exist, adding climate-resilient features may be seen as incremental. This highlights how the baseline of adaptation and the context where it is implemented can shape what is considered transformational or incremental. Without a multilaterally agreed-upon definition, some countries fear that Global North institutions will have the upper hand in de facto defining and operationalizing the concept^12^. Donors may also be hesitant to invest in a concept without a clear implementation roadmap.

Second, a major concern is that the debates on transformational adaptation might impose additional conditionalities to access international climate finance, making it even more difficult to mobilize finance and implement adaptation actions. In many places, inadequate financial resources, technology, and capacity already constrain adaptation. Some thus raise the question as to why calls for transformational adaptation are being made when international support for implementing adaptation already remains limited. Raising the bar to transformational adaptation also raises questions about the need to increase the means of implementation to support it.

Third, recognizing transformational adaptation as a distinct category of adaptation may also increase future reporting burdens, particularly under the already resource-intensive UNFCCC reporting processes. This could require additional indicators, methodologies, and capacity-building efforts to distinguish transformational adaptation from incremental measures, placing further demands on countries with limited resources. These arguments are particularly echoed in the context of the United Arab Emirates Framework for Global Climate Resilience, where indicators are developed to track progress towards the Global Goal on Adaptation.

Fourth, there are also countries that stress the need for context and placed-based approaches, raising concerns as to why this global discourse of transformational adaptation is being pushed instead of other nationally determined approaches. In academic literature, there are also many attributes attached to transformational adaptation, which some countries view as primarily domestic issues, such as strengthening climate justice and promoting inclusivity.

A final and much less explicitly voiced argument is around the idea that introducing and focusing international adaptation debates on ‘transformational adaptation’ is intentionally done to divert attention away from ongoing debates that are more relevant to some parties, including debates around ensuring Means of Implementation for adaptation, Loss and Damage mechanisms, or mitigation commitments from global north countries more generally.

## Navigating political realities in upcoming climate negotiations

Transformational adaptation as a concept has recently entered the political arena, and diverse views and opposition are to be expected as countries anticipate its implications. In a time when political trust among parties is already low^13^, new concepts, even when introduced with the best of intentions, can further strain already deadlocked negotiations. The political contestations around transformational adaptation are not about its underlying principles—there is broad agreement that more effective adaptation is urgently needed—but rather about the political and practical consequences it entails, including financial implications and increased reporting burdens.

To move the negotiations forward, scientific efforts can help clarify what transformational adaptation entails and how it can be achieved, identify points of convergence over when, where and why it might be needed, and propose concrete indicators to assess progress towards it. Concrete examples of what it looks like in practice can also play a powerful role in moving the conversations forward. Stories and cases that illustrate how communities, regions, or sectors are rethinking deeply embedded systems in response to climate risks can help ground abstract debates and spark more productive engagement^14^. However, at the heart of the matter are not just questions of knowledge and definitions, but also deeply rooted differences in worldviews, priorities, and interpretations of climate risks.

What is therefore urgently needed is a space for dialogue where countries can exchange views openly, explore how they understand and frame transformational adaptation, and acknowledge the political and practical implications of the concept. There are different arenas where such debates can take place. Within the UNFCCC, one option is to integrate transformational adaptation into the guiding questions for the Baku high-level dialogue on adaptation at each COP session. This dialogue is at the ministerial level, which might – at this stage – not be the most suitable place to exchange views openly. Given current debates, a more realistic option is to house a dialogue on transformational adaptation under the Baku Adaptation Road Map. Parties could establish an open-ended dialogue on transformational adaptation, using innovative formats that engage researchers, practitioners, and other non-state actors. This would encourage knowledge exchange across contexts and help build a shared understanding of the concept, its requirements, and implications. Over time, such dialogue could produce practical resources such as guidelines for integrating transformational adaptation across scales.

Beyond the UNFCCC, dialogue on transformational adaptation could also be facilitated, potentially under the leadership of a network of academic institutions. Ensuring the participation of UNFCCC negotiators is crucial for these dialogues to meaningfully contribute to future negotiation processes. Moreover, a key consideration would be to ensure regional balance, especially across global north and global south lines, and from governments, academia and practitioners.

Clearly these exchanges do not require immediate agreement, but they can help build trust and mutual understanding^15^. Disagreements and different viewpoints should not be seen as a failure, but as a natural and potentially constructive part of negotiating change. Any dialogue – within or outside the UNFCCC—should reflect on the first-order question of whether the concept of transformational adaptation is helping to advance climate action on the ground. In the end, the specific terminology may be less important than the willingness to engage with difficult questions about what effective, fair, and ambitious adaptation should look like.

## Data Availability

No datasets were generated or analysed during the current study.
